# Niclosamide induces protein ubiquitination and inhibits multiple pro-survival signaling pathways in the human glioblastoma U-87 MG cell line

**DOI:** 10.1371/journal.pone.0184324

**Published:** 2017-09-06

**Authors:** Benxu Cheng, Liza Doreen Morales, Yonghong Zhang, Shizue Mito, Andrew Tsin

**Affiliations:** 1 Department of Biomedical Science, School of Medicine, University of Texas Rio Grande Valley, Edinburg, Texas, United States of America; 2 South Texas Diabetes and Obesity Institute, School of Medicine, University of Texas Rio Grande Valley, Edinburg, Texas, United States of America; 3 Department of Chemistry, University of Texas Rio Grande Valley, Edinburg, Texas, United States of America; Duke University School of Medicine, UNITED STATES

## Abstract

Glioblastoma is the most common and lethal malignant primary brain tumor for which the development of efficacious chemotherapeutic agents remains an urgent need. The anti-helminthic drug niclosamide, which has long been in use to treat tapeworm infections, has recently attracted renewed interest due to its apparent anticancer effects in a variety of *in vitro* and *in vivo* cancer models. However, the mechanism(s) of action remains to be elucidated. In the present study, we found that niclosamide induced cell toxicity in human glioblastoma cells corresponding with increased protein ubiquitination, ER stress and autophagy. In addition, niclosamide treatment led to down-regulation of Wnt/β-catenin, PI3K/AKT, MAPK/ERK, and STAT3 pro-survival signal transduction pathways to further reduce U-87 MG cell viability. Taken together, these results provide new insights into the glioblastoma suppressive capabilities of niclosamide, showing that niclosamide can target multiple major cell signaling pathways simultaneously to effectively promote cell death in U-87 MG cells. Niclosamide constitutes a new prospect for a therapeutic treatment against human glioblastoma.

## Introduction

Glioblastoma multiforme is the most common and aggressive brain tumor (World Health Organization grade IV) for which an effective pharmacotherapy remains unavailable. The current initial treatment combines surgery with chemotherapy, yet the overall survival rate for glioma has not significantly improved in the past three decades because these tumors have a high incidence of recurrence and commonly lead to death within less than a year from diagnosis [1–3]. Extensive research has been done to identify more effectual antitumor regiments. Many efforts have been made to screen small molecular inhibitors against gliomas, however, first-generation inhibitors that selectively disrupt single targets or block a specific signaling pathway have failed to demonstrate clinical benefit in most patients with gliomas due to chemoresistance against antitumor treatments [4–6]. The mechanisms that lead to chemoresistance, which account for the limited efficacy of current glioma therapies, are not fully understood. Therefore, the development of new, more effective approaches that act through basic molecular mechanisms is critical to improve the prognosis for this type of tumor. One strategy to improve anti-cancer treatment and/or circumvent chemoresistance is to simultaneously disrupt multiple known oncogenic signaling pathways using either multiple single-target or multi-target therapeutics. It has been reported that up-regulation of the PI3K/AKT and MAPK/ERK pathways is involved in glioma tumorigenesis and aberrant tumor growth [7]. In addition, the well-known oncogene STAT3, a member of the STAT (signal transducers and activators of transcription) family that is de-regulated in a variety of cancers, is also important in glioblastoma tumorigenesis, as evidenced by the facts that STAT3 is activated in a high percentage of glioblastomas and its activation is associated with tumor grade and poor prognosis [8–10]. It has been suggested that controlling pro-survival signaling pathways as well as other molecular targets like STAT3 may represent a novel and effective therapeutic strategy for the treatment of gliomas [11]. Identification of a single multi-target agent that is already safely used by patients would be ideal seeing as it could be quite potent against aggressive tumors and it could be more quickly implemented in cancer treatment.

Niclosamide, an FDA approved oral anti-helminthic drug, has been used for nearly 50 years to treat most tapeworm infections due to its efficacy in inhibiting mitochondrial oxidative phosphorylation and anaerobic adenosine triphosphate (ATP) production [12]. Studies in the past few years have demonstrated that niclosamide is a promising chemotherapeutic agent. A number of research groups have reported that niclosamide had potent anti-proliferative activity and induced cytotoxicity in a broad spectrum of cancer cells including solid tumor cells, e.g. head and neck cancer [13]; non-small cell lung cancer [14]; prostate cancer [15]; colon cancer [16]; ovarian cancer [17–18]; acute myelogenous leukemia (AML) [19]; osteosarcoma [20]; and breast cancer [21–22]. It has been shown that niclosamide effectively limits different types of cancer growth both *in vitro* and *in vivo* by triggering apoptosis and oxidative stress or by inhibiting several important signaling pathways including Wnt/β-catenin [23–25], mTOR [26], NFκB [19], and STAT3 [14,27]. Intriguingly, it was also discovered that niclosamide stimulates autophagy, an intracellular process by which unessential or ineffectual cytoplasmic components are degraded [26]. Recently, a study of primary human glioblastoma cells identified niclosamide as a potential anticancer agent against glioblastoma given that it demonstrated cytotoxic and anti-migratory effects [28]. However, studies exploring putative anticancer properties in glioma cells are limited, and the molecular mechanisms underlying its effects remain poorly understood. Therefore, the present study aims to describe anti-glioblastoma properties of niclosamide in the human U-87 malignant glioma (MG) cell line.

In this study, we observed that niclosamide inhibits U-87 MG cell proliferation and it induces accumulation of ubiquitinated proteins, apoptosis, ER stress, autophagy, and cell death. Furthermore, we found that niclosamide not only inhibits STAT3 and Wnt/β-catenin signaling, which has been seen in other cancer cells, but it also down-regulates two other pro-survival signal pathways (PI3K/AKT and MAPK/ERK) in these cells. These findings show that niclosamide is a putative candidate for use in brain cancer treatment and understanding the mechanism of action of niclosamide will facilitate the development of more effective chemotherapeutics.

## Materials and methods

### Reagents

Niclosamide and autofluorescent agent Monodansylcadaverine (MDC) were purchased from Sigma-Aldrich (St. Louis, MO). Niclosamide was dissolved in DMSO at a 10 mM concentration and stored at –20°C.

### Cell culture

The human glioblastoma U-87 MG cell line was purchased from the American Type Culture Collection (ATCC HTB-14, Manassas, VA). Cells were cultured at 5% CO_2_ at 37°C in DMEM supplemented with 10% fetal bovine serum (FBS), penicillin (100 units/mL), and streptomycin (100 μg/mL). Cells were sub-cultured weekly onto 60 mm or 100 mm tissue culture dishes and used for experiments at 85–90% confluence. The cell culture medium was replaced every 2–3 days.

### Cell viability assays

MTS assay [3-(4,5-dimethylthiazol-2-yl)-5-(3-carboxyme-thoxyphenyl)-2-(4-sulfophenyl)-2H-tetrazolium, inner salt] was performed in 96-well plates using a CellTiter 96 non-radioactive cell proliferation colorimetric assay kit (Promega, Madison, WI) according to manufacturers' instructions. Briefly, cells (8,000 cells in 200 μl medium per well) were plated in 96-well plates the day before the experiment. At the end of various treatments, 100 μl medium was removed from well, followed by the addition of 20 μl of MTS solution to each well and then incubated for 1 h at 37°C. Samples were read by a microplate reader at a wavelength of 490 nm. At least 6 replicates of each treatment were used.

### Monodansylcadaverine (MDC) staining

The autofluorescent agent MDC (Sigma-Aldrich) was used as a specific autophagolysosome marker to analyze the autophagic process [29]. U-87 MG cells were seeded on glass-bottom slides in growth medium and incubated overnight. Cells were treated with or without 5 μM niclosamide for 24 h. The cells were incubated with 0.05 mM MDC for another 1 h at 37°C, and then washed four times with PBS (pH 7.4). Cells were immediately visualized and imaged by confocal microscopy.

### Western blot analysis

Changes in the amounts of protein expression were measured by Western blot analysis. Protein homogenates were prepared as follows: the cells were lysed in ice-cold RIPA lysis buffer containing protease and phosphatase inhibitor cocktails (Santa Cruz Biotechnology, Santa Cruz, CA). Clear lysates were obtained by centrifugation at 4°C for 20 min at 13,000 rpm in a refrigerated microcentrifuge. Protein concentrations were determined, according to the manufacturer’s instruction, using the Pierce BCA Protein Assay Kit (Pierce, Rockford, IL, USA). Equal amounts of the protein samples (25–30 μg) were separated on a 10% or 4–20% gradient polyacrylamide gel (Bio-Rad Laboratories, Hercules, CA), transferred to nitrocellulose membranes, and blocked for either 1 h at room temperature or overnight at 4°C with Tris buffer containing 0.1% Tween 20 (TBS-T, pH 7.4) and 5% (w/v) nonfat dried milk. The blotted membranes were incubated with specific primary antibodies for 1 h at room temperature or overnight at 4°C. Ubiquitin, cyclin D1, survivin, P-ERK and ERK antibodies were purchased from Santa Cruz Biotechnology while P-AKT, AKT, CHOP, LC3, Cleaved PARP, and cleaved caspase-3 antibodies were purchased from Cell Signaling (Beverly, MA). β-Actin loading control was obtained from Sigma-Aldrich. The membranes were washed and incubated for 1 h with appropriate horseradish peroxidase-conjugated secondary antibodies. The protein bands were detected using a chemiluminescent (ECL) method, according to the manufacturer’s instructions. Band densities were analyzed by ImageJ software.

### Statistical analysis

Statistical analysis was undertaken using one-way analysis of variance and Tukey comparison test, using GraphPad Prism 4.0 (GraphPad Software). Data is presented as the mean ± SEM of at least three independent experiments. In the figures, asterisks indicate the degree of significance difference (*p≤0.05, ***p≤0.001) between different treated cell cultures in comparison to untreated controls.

## Results

### Niclosamide promotes apoptosis by protein ubiquitination

**Fig 1:**
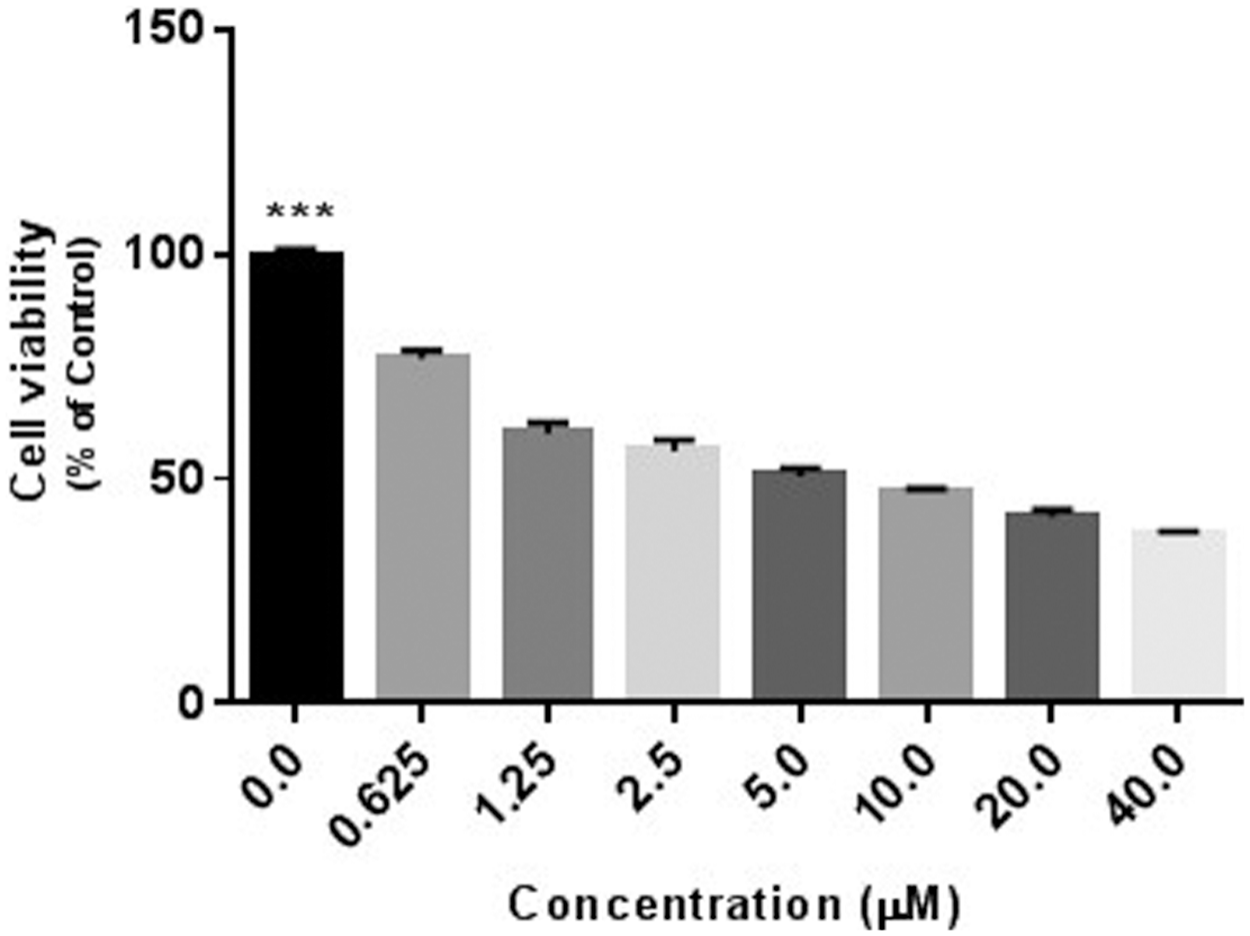
Niclosamide treatment reduces U-87 MG cell viability. U-87 MG cells were treated with the indicated concentrations of niclosamide for 24 h. Cell viability was determined by MTS assay. Data represent the mean ± S.E.M of at least three independent experiments. (***) *p*<0.001.

Dysregulation of apoptosis, or programmed cell death, is a hallmark of cancer as it is a critical cellular process by which damaged or abnormal cells are eliminated. Niclosamide has been shown to promote apoptosis in various cancer cells including U-87 MG cells [28]. To confirm this effect against U-87 MG cells, cells were treated with increasing concentrations of niclosamide (0–40μM) for 24 hours. MTS assay was used to measure cell proliferation and viability as described in the Material and Methods. As shown in Fig 1, niclosamide significantly inhibits cell proliferation/viability in a dose-dependent manner.

**Fig 2:**
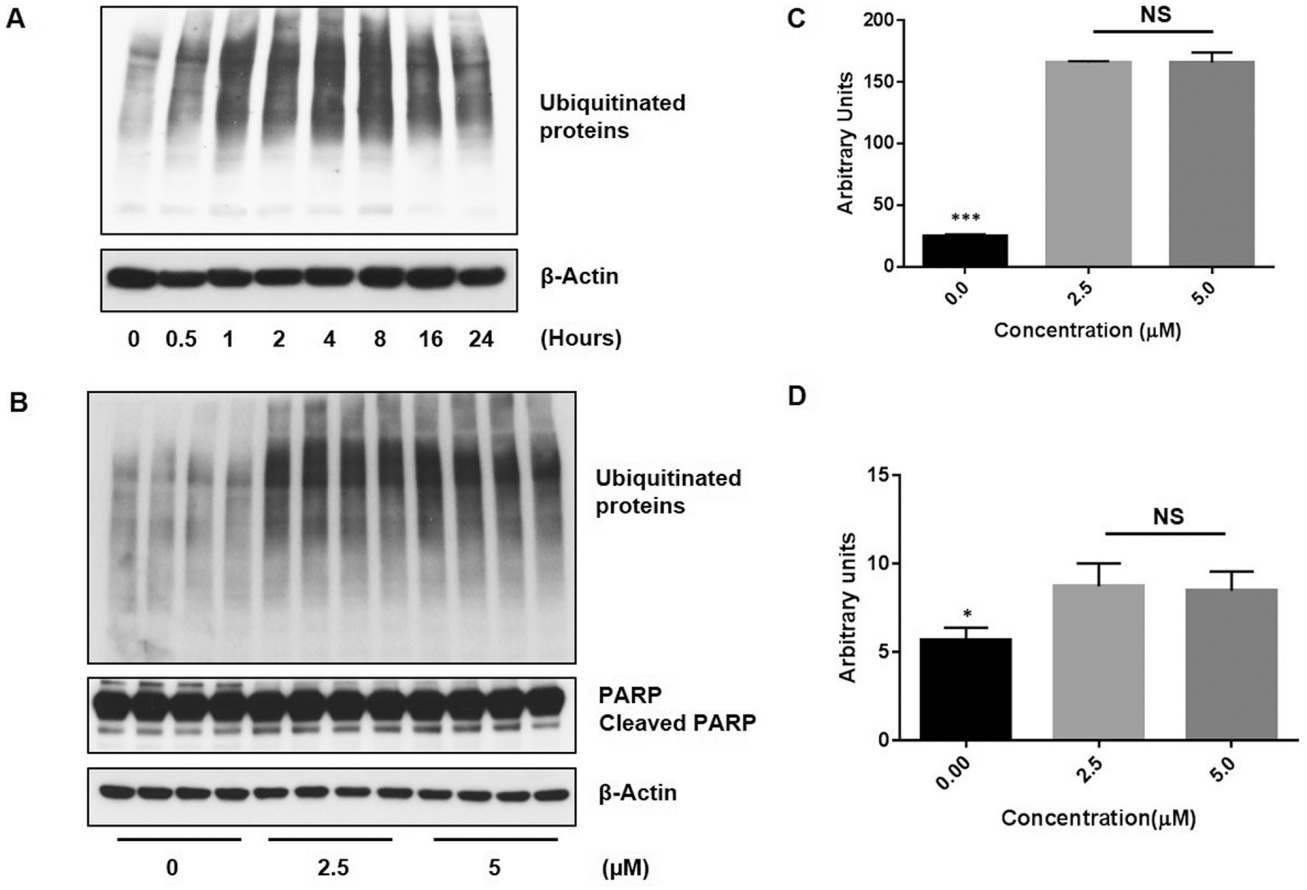
Niclosamide promotes protein ubiquitination and apoptosis in U-87 MG cells. (A) U-87 MG cells were treated with 5 μM niclosamide and cells were collected at the indicated time points. Total cell lysates were resolved by SDS-PAGE and immunoblotted with antibody specific to ubiquitin. (B) Total cell lysates were isolated from U-87 MG cells treated with the indicated concentrations of niclosamide for 24 h, resolved by SDS-PAGE and then immunoblotted with antibody specific for ubiquitin and PARP, an apoptotic protein. β-Actin was utilized as a loading control. The image is representative of at least three independent experiments. Relative expression levels of ubiquitinated proteins and cleaved PARP protein from cells with and without niclosamide treatment were quantified by densitometry (C and D, respectively). Data represent the mean ± S.E.M of at least three independent experiments. (***) *p*<0.01 and (*) *p*<0.05, respectively; NS = no significant difference.

The ubiquitin-proteasome system (UPS) is another important cellular signal transduction pathway necessary for the degradation of intracellular proteins and it can play a role in the regulation of pathways necessary for tumor cell growth and survival. Studies have shown that ubiquitination also can play a role in the regulation of apoptosis [30–31]. A previous study has shown that niclosamide was able to prevent the formation of large ubiquitin-containing aggregates caused by proteasome inhibition in human neuroblastoma SH-SY5Y cells by selectively targeting proteins for degradation *via* the lysosomes or another proteasome-independent pathway [32]. Therefore, we investigated whether or not exposure to niclosamide could also suppress protein ubiquitination in U-87 MG cells. Ubiquitinated proteins were detected by Western blot analyses with ubiquitin antibody (Fig 2). Remarkably, treatment with niclosamide resulted in an increase in the level of ubiquitinated proteins, suggesting niclosamide can trigger the accumulation of ubiquitin-containing aggregates in U-87 MG cells. As shown in Fig 2A, an increase in protein ubiquitination could be observed within 30 minutes of exposure to niclosamide in comparison to untreated control and remained elevated thereafter. Detection of ubiquitinated proteins appeared to decrease slightly after 16 hours, possibly due to partial protein degradation by the UPS. To assess whether or not the increase of abundant protein ubiquitination following treatment with niclosamide is specific to U-87 MG cells, an additional human glioblastoma cell line (U-118 MG), one fibroblast cell line, one osteosarcoma cell line and two breast cancer cell lines were treated with different concentrations of niclosamide for 24 h. Western blot analysis of U-118 MG cells showed similar results to U-87 MG cells (S1 Fig). Exposure to niclosamide resulted in a substantial increase of protein ubiquitination. In contrast, western blot analysis showed no obvious change in ubiquitinated protein levels between treated and untreated controls in the other tested cell lines (S2 Fig). The effect of niclosamide on protein ubiquitination has not previously been reported for any human glioblastoma cell line and the data suggests that the effect may be unique to human glioblastoma cells. We also examined whether or not the increase of ubiquitinated proteins following niclosamide treatment is caused by proteasome inhibition. Niclosamide had no significant effect on proteasome activity in U-87 MG cells (S2 Fig) as measured by a 20S proteasome activity assay [33].

Finally, cells were treated with 2.5 μM or 5 μM niclosamide for 24 hours and Western blot analysis was performed with ubiquitin antibody and PARP (Poly ADP ribose polymerase) antibody, a well-known and commonly used marker for apoptosis. As shown in Fig 2B and 2C, treatment with niclosamide resulted in a significant increase in expression of ubiquitinated proteins. Cleaved PARP expression also significantly increased in U-87 MG cells following exposure to niclosamide (Fig 2B and 2D), which is consistent with previous results in primary human glioblastoma cells [28] and with our results demonstrating that niclosamide inhibits cell viability. Given that reduced cell viability and increased PARP expression were concomitant with an increased level of ubiquitinated proteins, these results suggest that niclosamide may have induced apoptosis in U-87 MG cells by promoting protein ubiquitination.

### Niclosamide induces ER stress and activates autophagy

**Fig 3:**
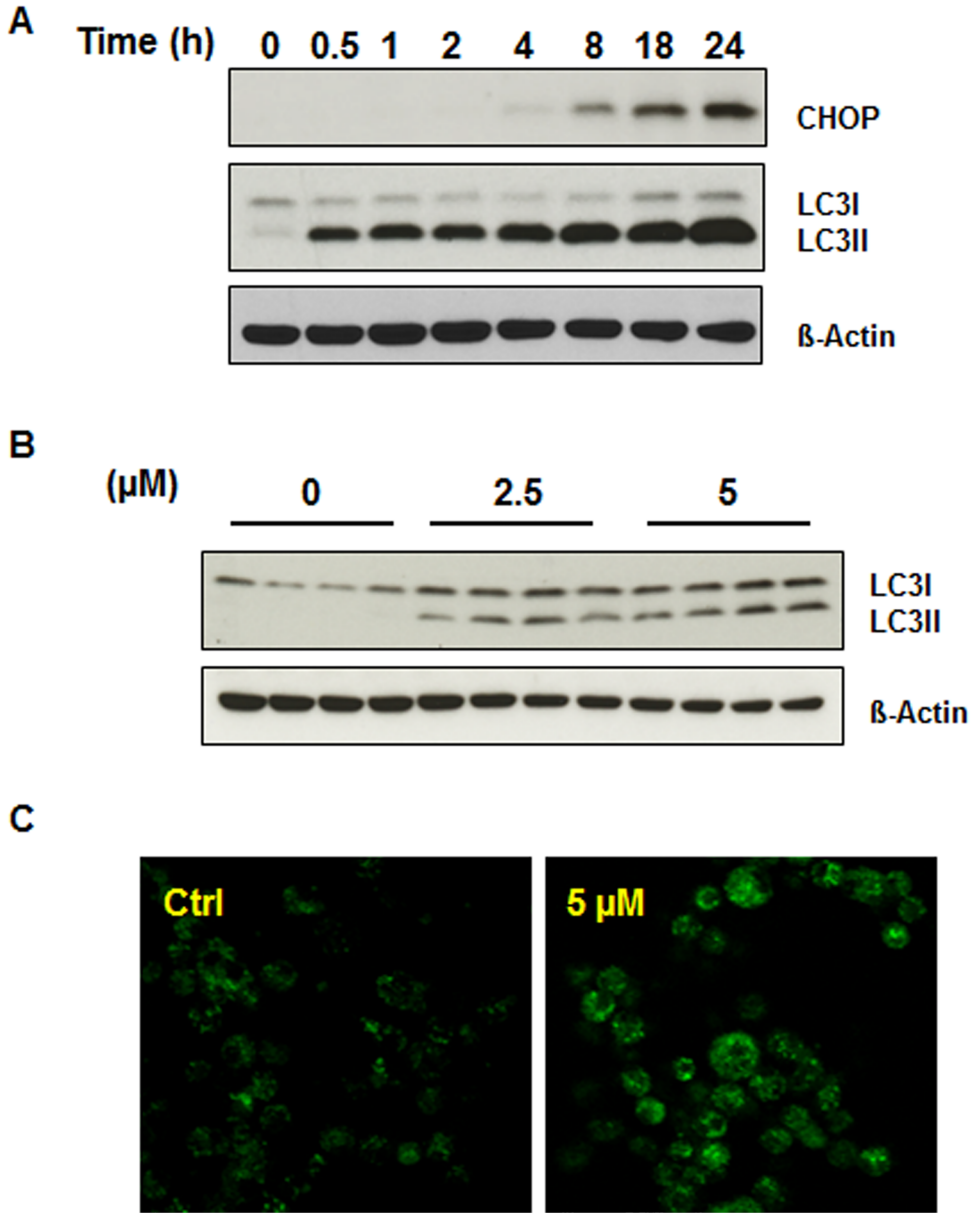
Niclosamide triggers ER stress and the autophagic response in U-87 MG cells. (A) U-87 MG cells were treated with 5 μM niclosamide and collected at the indicated time points. Total cell lysates were resolved on SDS-PAGE and immunoblotted with antibodies specific for CHOP and LC3. (B) U-87 MG cells were treated with 2.5 or 5 μM niclosamide and total protein was isolated from lysed cells after 24 h. Lysates were resolved on SDS-PAGE and immunoblotted with LC3-specific antibody. (C) Representative image of U-87 MG cells incubated with 5 μM niclosamide for 24 h and then stained with MDC (0.05 mM). Fluorescence particles in the cytoplasm indicate autophagic vacuoles.

It has been demonstrated that accumulation of misfolded or ubiquitinated proteins can function like proteasome inhibition and induce endoplasmic reticulum (ER) stress [34–36]. ER stress is a cellular response to the disruption of ER function. Failure to eliminate aberrant proteins and regain ER homeostasis can lead to severe cellular stress which then triggers cell death. This event is mediated by specific pathways involving proteins such as CHOP/GADD153 (C/EBP homology protein or growth arrest and DNA damage inducible protein 153) [35,37]. ER stress is associated with a variety of human disease including cancer. To determine whether or not niclosamide treatment can stimulate ER stress, U-87 MG cells were treated with 5 μM niclosamide for various time periods up to 24 hours. Cells were then collected and cell lysates were utilized for Western blot analysis with CHOP antibody given that CHOP is a major ER stress-associated protein. As expected, niclosamide promoted accumulation of CHOP in a time-dependent manner (Fig 3A).

Recent studies have found that ER stress is linked to autophagy which can function as a protective mechanism during ER stress [35,38]. As niclosamide has also been reported to induce autophagy in human breast cancer MCF-7 cells [26], we next aimed to investigate whether niclosamide has any influence on autophagy in U-87 MG cells. First, cells were treated with 5 μM niclosamide and cell lysates were collected at different time points up to 24 hours. Western blot analysis was carried out using LC3 (light chain 3) antibody, a widely-used marker for autophagy. The conversion of cytosolic LC3-I to membrane-bound LC3-II indicates autophagic activity, specifically the formation of autophagosomes. As shown in Fig 3A, niclosamide caused a time-dependent increase in LC3-II protein levels in U-87 MG cells, indicating activation of autophagy. Next, cells were treated with different concentrations of niclosamide for 24 hours. Treatment with either 2.5 μM or 5 μM niclosamide resulted in expression of LC3-II and the autophagy response (Fig 3B). To further confirm that niclosamide induces autophagy, monodansylcadaverine (MDC), a fluorescent compound, was used to stain niclosamide-treated cells. As shown in the Fig 3C, abundant autophagic vacuoles appeared in the cytoplasm of cells exposed to 5 μM niclosamide for 24 hours, whereas a lesser amount of fluorescent dots could be observed in the cytoplasm of control (untreated) cells.

### Niclosamide inhibits Wnt/β-catenin signaling which can facilitate apoptosis

**Fig 4:**
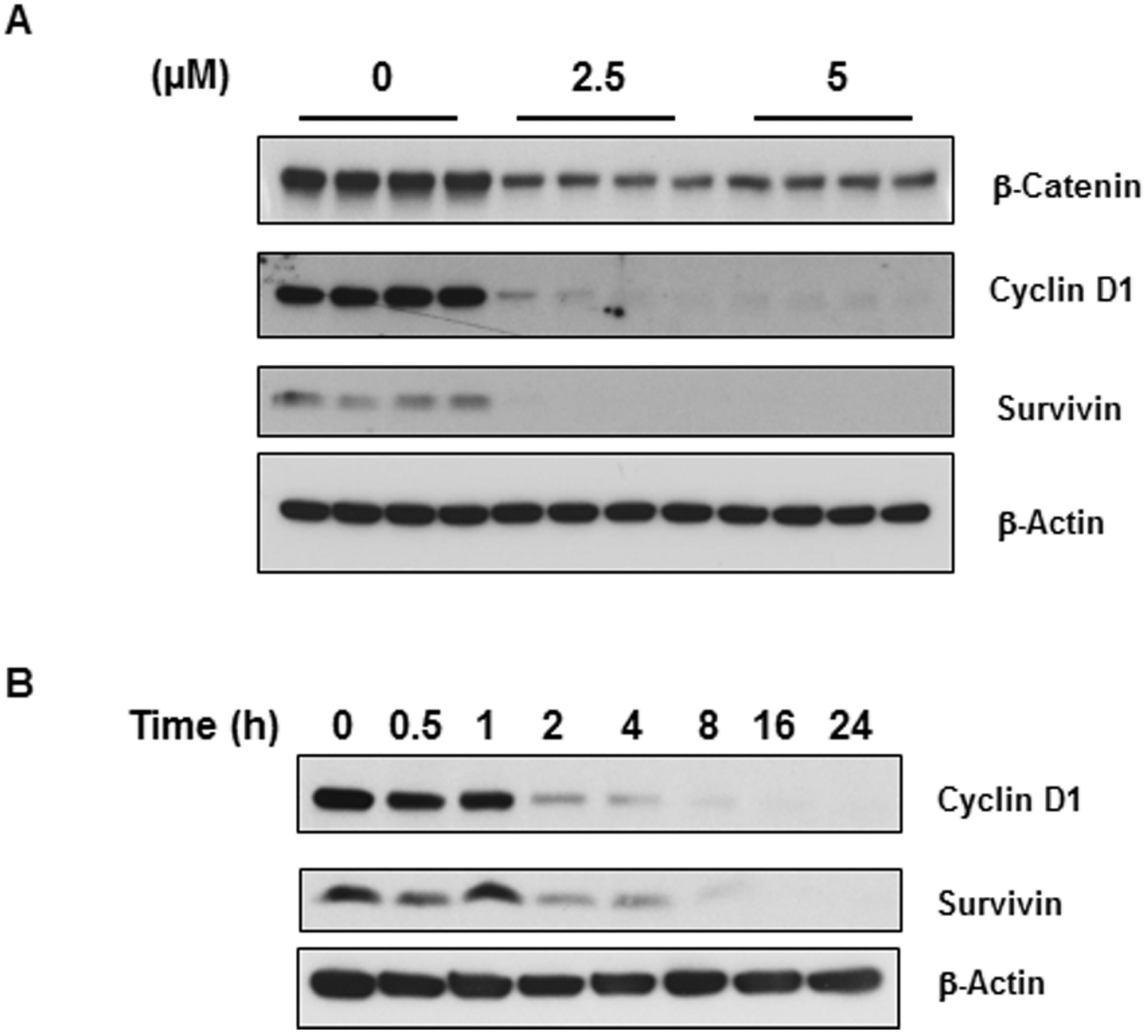
Niclosamide represses expression of β-catenin and its downstream effectors. (A) U-87 MG cells were treated with two levels of niclosamide as indicated in the figure for 24 h. Total protein isolated from cell lysates were resolved by SDS-PAGE and immunoblotted with antibodies specific for β-catenin and proteins from two target genes, cyclin D1 and survivin. β-Actin was used as a loading control. (B) Time course effect of niclosamide on the expression of cyclin D1 and survivin.

The Wnt/β-catenin signaling pathway is involved in cell proliferation, differentiation, and migration and it is required for neural stem cell development. Many studies have demonstrated that Wnt/β-catenin signaling is up-regulated in glioblastoma and can promote glioblastoma growth and invasion given its function in stem cell development [39]. Therefore, we wanted to analyze the effect of niclosamide on Wnt/β-catenin signaling in U-87 MG cells by Western blot analysis. Cells were treated with two levels of niclosamide (2.5 μM or 5 μM). As shown in Fig 4A, β-catenin expression levels were markedly decreased after exposure to niclosamide for 24 hours, suggesting that niclosamide has an inhibitory effect on Wnt/β-catenin signaling. We then examined the expression of two β-catenin downstream targets, survivin and cyclin D1, in U-87 MG cells. Cyclin D1 is a critical protein required for cell cycle progression whereas survivin negatively regulates apoptosis by inhibiting caspase activation. Similar to β-catenin, both cyclin D1 and survivin expression were significantly suppressed after 24-hour treatment with niclosamide (Fig 4A). Furthermore, their expression levels decreased in a time-dependent manner (Fig 4B). These results suggest that niclosamide inhibits cell cycle progression, reduces cell viability, and facilitates apoptosis in U-87 MG cells by inhibiting Wnt/β-catenin signaling.

### Niclosamide inhibits PI3K/AKT and MAPK/ERK pro-survival signal transduction

**Fig 5:**
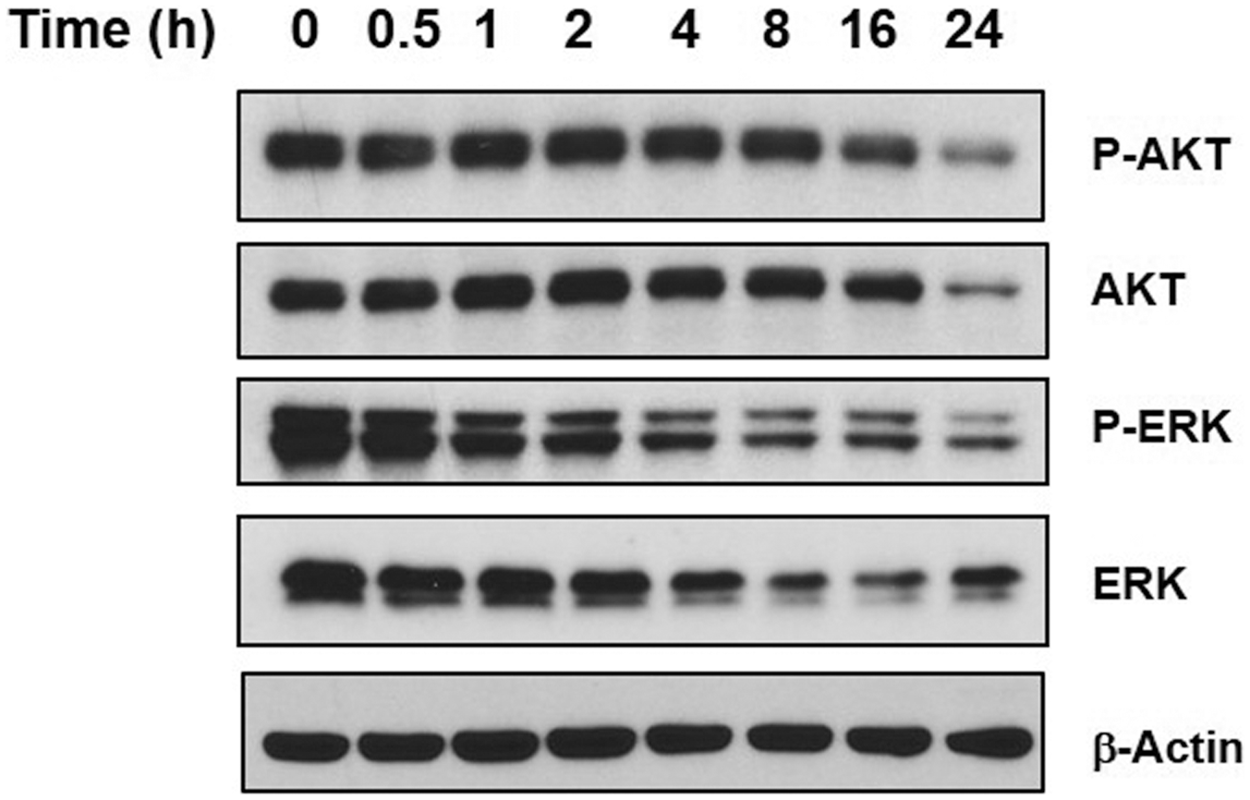
Niclosamide suppresses AKT and ERK expression in U-87 MG cells. U-87 MG cells were treated with 5 μM niclosamide and cells were collected at time points indicated in the figure. Total protein was isolated from treated cells, resolved by SDS-PAGE and immunoblotted with specific antibodies against P-AKT, AKT, P-ERK and ERK. β-Actin was used as a loading control.

The PI3K/AKT pathway is an intracellular signaling cascade important in regulating the cell cycle and it is directly related to cellular proliferation and cancer development. The MAPK/ERK signaling pathway is an evolutionarily conserved kinase module that links extracellular signals to cellular responses that control fundamental cellular processes such as proliferation, differentiation, and apoptosis. Given the importance of these two signal transduction pathways, they can often interconnect [40]. Previous studies have shown that niclosamide does not impair PI3K/AKT signaling and has little effect on the MAPK/ERK signaling pathway in MCF-7 cells [41]. However, we wished to evaluate whether or not niclosamide may have an effect on these signaling pathways in U-87 MG cells, Western blot analysis was conducted using phosphorylated/activated AKT, total AKT, phosphorylated/activated ERK, and total ERK antibodies (Fig 5). Surprisingly, niclosamide treatment resulted in a reduction of both phosphorylated AKT and total AKT over time which corresponds with the decrease in cell viability that we had observed after 24 h treatment. Niclosamide treatment also appeared to suppress both ERK phosphorylation and ERK expression in a time-dependent manner. The data suggests that niclosamide has an inhibitory effect on upstream regulators of AKT and ERK which subsequently affects their expression and activation and leads to disruption of AKT and ERK function on their downstream effectors.

### Niclosamide suppresses STAT3 to inhibit cell survival

**Fig 6:**
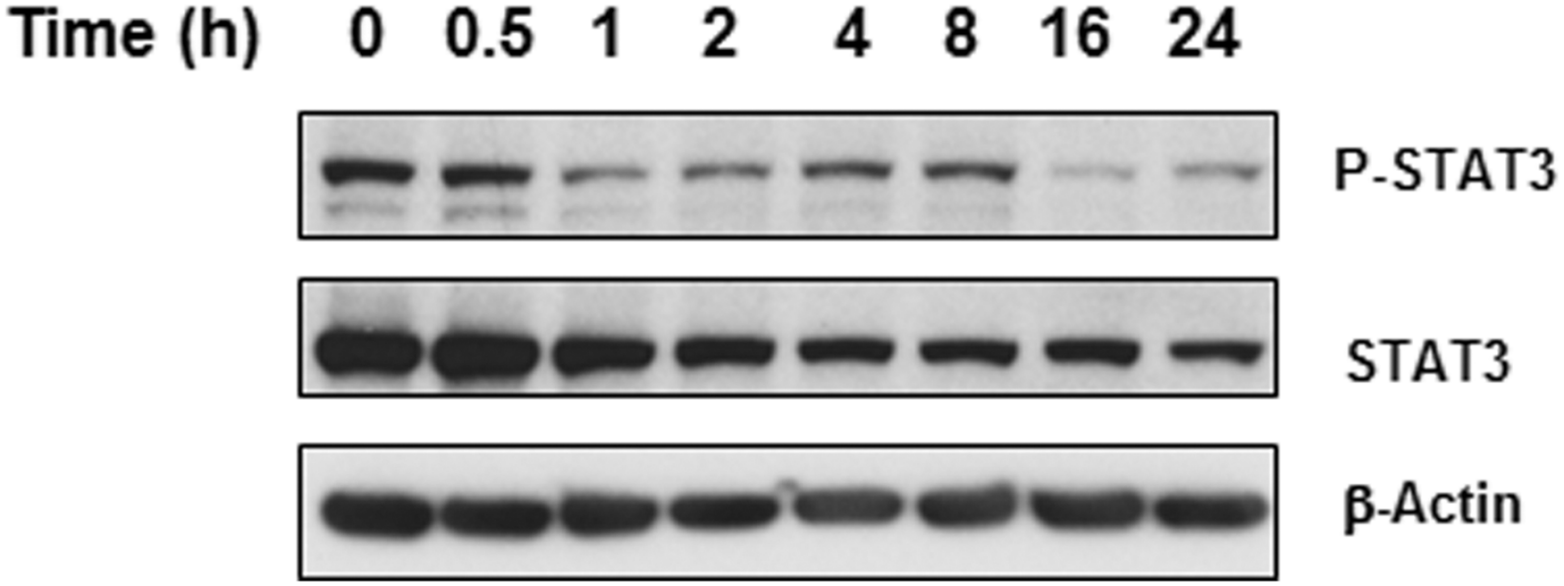
Niclosamide inhibits STAT3 expression. Total protein was isolated from U-87 MG cells treated with 5 μM niclosamide at the times indicated in the figure. Lysates were resolved by SDS-PAGE and immunoblotted with either P-STAT3 or STAT3-specific antibodies. β-Actin was used as loading control.

STAT3 regulates major cellular processes such as cell growth, survival and differentiation and, as previously mentioned, it is dysregulated in a number of cancers and thereby is intimately linked to tumorigenesis. Studies have demonstrated that niclosamide is a potential anticancer drug against cancers with activated STAT3 given that it can disrupt STAT3 transcription [14,27]. Also, evidence has shown that inhibition of STAT3 with niclosamide in lung cancer cells resulted in increased sensitivity to radiotherapy [42]. To determine whether or not niclosamide has any effect on the STAT3 signaling pathway in U-87 MG cells, we used immunoblotting for phosphorylated/activated STAT3 and total STAT3. Cells were treated with 5 μM niclosamide for different time periods up to 24 hours. As shown in Fig 6, niclosamide-treated cells showed a decrease in expression of both STAT3 and phosphorylated STAT3 in a time-dependent manner like ERK. Hence, niclosamide may inhibit cell proliferation of U-87 MG cells, in part, by mediating inhibition of STAT3 expression and activation.

## Discussion

Recent studies have shown that niclosamide is cytotoxic and, more importantly, it exhibits anticancer activity in different cancer cell lines. It has been demonstrated that, like many other anticancer reagents, niclosamide has an effect on various important cellular mechanisms involved in carcinogenesis. Niclosamide is able to inhibit signaling pathways such as Wnt/β-catenin, NF-κB (a regulator of inflammation), STAT3, and mTORC1 (a negative regulator of autophagy), depending on cancer cell type. However, the effect of niclosamide on human glioblastoma has not been thoroughly studied. Our study aimed to investigate the effect of niclosamide in the human glioma U-87 MG cell line. In the current study, we sought to evaluate the toxicity of niclosamide in U-87 MG cells. Our results are consistent with other published reports on the mechanisms of niclosamide-induced apoptotic cell death in other model systems, suggesting that niclosamide may employ common cytotoxic signaling pathways in different cancer cell lines. Results presented in this study demonstrated that niclosamide dramatically inhibits U-87 MG cell growth and proliferation, which correlates with results from a previous study [25]. Significant anti-proliferative actions could be detected at tested concentrations from 0.625–40 μM (Fig 1) in a dose-dependent manner.

Previous studies have shown controversial results regarding the effect of niclosamide on protein ubiquitination. It was reported that niclosamide was able to prevent the formation of large ubiquitin-containing aggregates in human neuronal SH-SY5Y cells [32]. However, this finding is contrary to some recent reports showing that niclosamide increases formation of Tax-ubiquitinated aggregates and protein ubiquitination of β-catenin [19,43]. Hence, we attempted to test any effect of niclosamide on protein ubiquitination in U-87 MG and U-118 MG cells. Surprisingly, exposure of U-87 MG cells or U-118 MG cells to niclosamide tremendously induced protein ubiquitination (Fig 2 and S1 Fig, respectively) without significant change to proteasome activity (S3 Fig). For the first time, we demonstrate that niclosamide is capable of producing pronounced protein ubiquitination in human glioma U-87 MG and U-118 MG cells at a level similar to that induced by proteasome inhibitors, agents that have been extensively studied for their potential potent anticancer properties. This result implies that the accumulation of ubiquitin-containing protein aggregates in glioblastoma cells may suppress cell proliferation and trigger apoptosis. Indeed, our Western blot analysis shows that niclosamide induces apoptosis in U-87 MG cells (Fig 2B). Xiang et al. reported that niclosamide down-regulated the retroviral oncoprotein Tax in HTLV-1-transformed T cell by promoting the formation of polyubiquitinated Tax protein aggregate, facilitating its subsequent degradation in the proteasome [43]. Similarly, recent studies on chronic myelogenous leukemia (CML) cells noted that the ubiquitination of β-catenin was increased following niclosamide treatment [19]. It is very interesting that niclosamide stimulated protein ubiquitination in the U-87 MG (Fig 2) and U-118 MG (S1 Fig) glioblastoma cell lines, whereas it had no effect on protein ubiquitination in other human cell lines (S2 Fig). These results are indicative of niclosamide-induced protein ubiquitination being unique to human glioblastoma cells. It is still unclear whether or not the increase in protein ubiquitination seen in U-87 MG and U-118 MG cells only applies to specific proteins and we want to confirm that induction of protein ubiquitination results in the formation of large ubiquitin-containing aggregates. In the future, we plan to more fully characterize the exact underlying mechanisms that allow niclosamide to induce protein ubiquitination in glioblastoma by utilizing more glioblastoma cell lines.

Another important outcome of this research was the demonstration, for the first time, that niclosamide induces endoplasmic reticulum (ER) stress in U-87 MG cells. We found that U-87 MG cells exposed to niclosamide showed increased expression of CHOP, a key marker of ER stress. Moreover, our results showed that niclosamide is able to induce ER stress in a time-dependent manner. Thus, the ability of niclosamide to trigger ER stress in U-87 MG cells may be one mechanism by which niclosamide can trigger cell death of cancer cells and thus function as an effective anticancer agent.

Previous results have shown that niclosamide is able to induce autophagy in various cancer cells including human neuroblastoma SH-SY5Y cells. Balgi et al. demonstrated that niclosamide induces autophagosome formation and inhibits mTORC1 signaling in MCF-7 breast cancer cells [26]. Niclosamide also can stimulate both apoptotic and autophagic cell death in HeLa cells [44]. Gies’s group demonstrated that in SH-SY5Y cells niclosamide prevents MG132-mediated ubiquitin-containing aggregates by inducing autophagy [32]. Furthermore, ER stress can induce autophagy in order to reestablish homeostasis [34,36] and we observed niclosamide triggered ER stress in U-87 MG cells (Fig 3A). Therefore, we examined the effect of niclosamide on autophagic activation in U-87 MG cells. Consistent with the previous studies on other cell lines, we observed that following niclosamide treatment there was an increase of autophagy in U-87 MG cells as evidenced by an increase in LC3-II protein and an increase of MDC staining for autophagosomes in niclosamide treated cells (Fig 3B and 3C). The results suggest niclosamide elicits the autophagic response and this may occur, at least in part, through the activation of ER stress. Autophagy has been established as a mechanism that contributes to cell survival, however current investigations have provided strong evidence that, under certain conditions, autophagy also can promote apoptosis. For example, it has been reported that treatment with the antimalarial (and anticancer) drug chloroquine is able to trigger autophagy-mediated cell death of glioma cells [45]. Therefore, we hypothesize that the activation of autophagy by niclosamide may partially contribute to the initiation of cell death in glioma U-87 MG cells. Interestingly, niclosamide-induced activation of autophagy within U-87 MG cells corresponded with a rise in protein ubiquitination (Fig 2) whereas in SH-SY5Y cells the niclosamide-mediated autophagic response led to inhibition of protein ubiquitination. Research has shown that there are links between the UPS and autophagy as they have similar substrates and regulators, such as histone deacetylase 6 (HDAC6) and p62, with ubiquitin being one key to targeting proteins for degradation by either system [46–47]. Further study is needed to characterize the underlying mechanism(s) that result in the different responses to niclosamide by U-87 MG cells and SH-SY5Y cells.

The Wnt signaling pathway plays a pivotal role in human cancer cell growth and carcinogenesis. Multiple publications by various groups have shown that niclosamide is a potent inhibitor of Wnt/β-catenin signaling. In particular, niclosamide has been reported to inhibit Wnt/β-catenin signaling in various cancers [23–25,48]. Our data demonstrated that niclosamide is able to down-regulate the expression of Wnt/β-catenin and proteins from their target genes, cyclin D1 and survivin, in U-87 MG cells as well. The expression of cyclin D1 and survivin significantly decreased in a concentration-dependent manner which correlated with the results of β-catenin (Fig 4) and phospho-STAT3 expression (Fig 6). The cyclin D1 proto-oncoprotein is a crucial regulator in cell-cycle progression, and aberrant overexpression of cyclin D1 is linked to tumorigenesis of many different cancer types [49].

Both PI3K/AKT and MAPK/ERK signaling pathways have been reported to play an important role in the tumorigenesis of glioma cells [50–51]. The PI3K/AKT signaling pathway exerts a critical role in the promotion of cell survival and the inhibition of apoptosis in cancer cells, especially glioblastoma cells [52–53]. Previous studies have demonstrated that niclosamide does not impair PI3K/AKT signaling in human breast cancer MCF-7 cells [41], and it has no effect on this pathway in the human prostate cancer cell line DU145 nor in human glioma cell line A172 [54]. However, our findings demonstrate that niclosamide is able to inhibit PI3K/AKT and MAPK/ERK signaling in human glioma U-87 MG cells, which, to the best of our knowledge, has not been reported in any previous cancer studies. We clearly see a decrease in AKT and ERK activation following niclosamide treatment (Fig 5). The results indicate that the inhibition of pro-survival PI3K/AKT and MAPK/ERK signaling pathways may enhance the effect of niclosamide-induced cell death and thereby niclosamide may be a novel effective anticancer agent against grade IV glioblastoma like the U-87 MG cell line. Again, further study utilizing additional glioblastoma cell lines is required.

Niclosamide has also recently been reported as a potent STAT3 inhibitor that functions as a tumor suppressor in cancer cells [27]. In agreement with previous studies, we found that both STAT3 and phospho-STAT3 protein expression in U-87 MG cells was dramatically reduced upon exposure to niclosamide (Fig 6). MAPK/ERK signaling is one of the many pathways that is capable of regulating STAT3 *via* phosphorylation [55]. Therefore, suppression of ERK by niclosamide may partially contribute to inhibition of STAT3. STAT3 plays an important role in anti-apoptotic signaling within various cancer cells [9,56]. High levels of STAT3 activity have been found to predict intrinsic chemotherapy resistance and STAT3 inhibition allows glioblastoma cells to overcome temozolomide resistance which is a significant finding given that temozolomide is a first-line treatment for glioblastoma [2,57]. Furthermore, STAT3 is required for proliferation and maintenance of multipotency in glioblastoma stem cells [56]. Our results imply that niclosamide-mediated inhibition of STAT3 may contribute to the overall efficacy of niclosamide as a tumor suppressor to repress U-87 MG cell proliferation and promote cell death.

**Fig 7:**
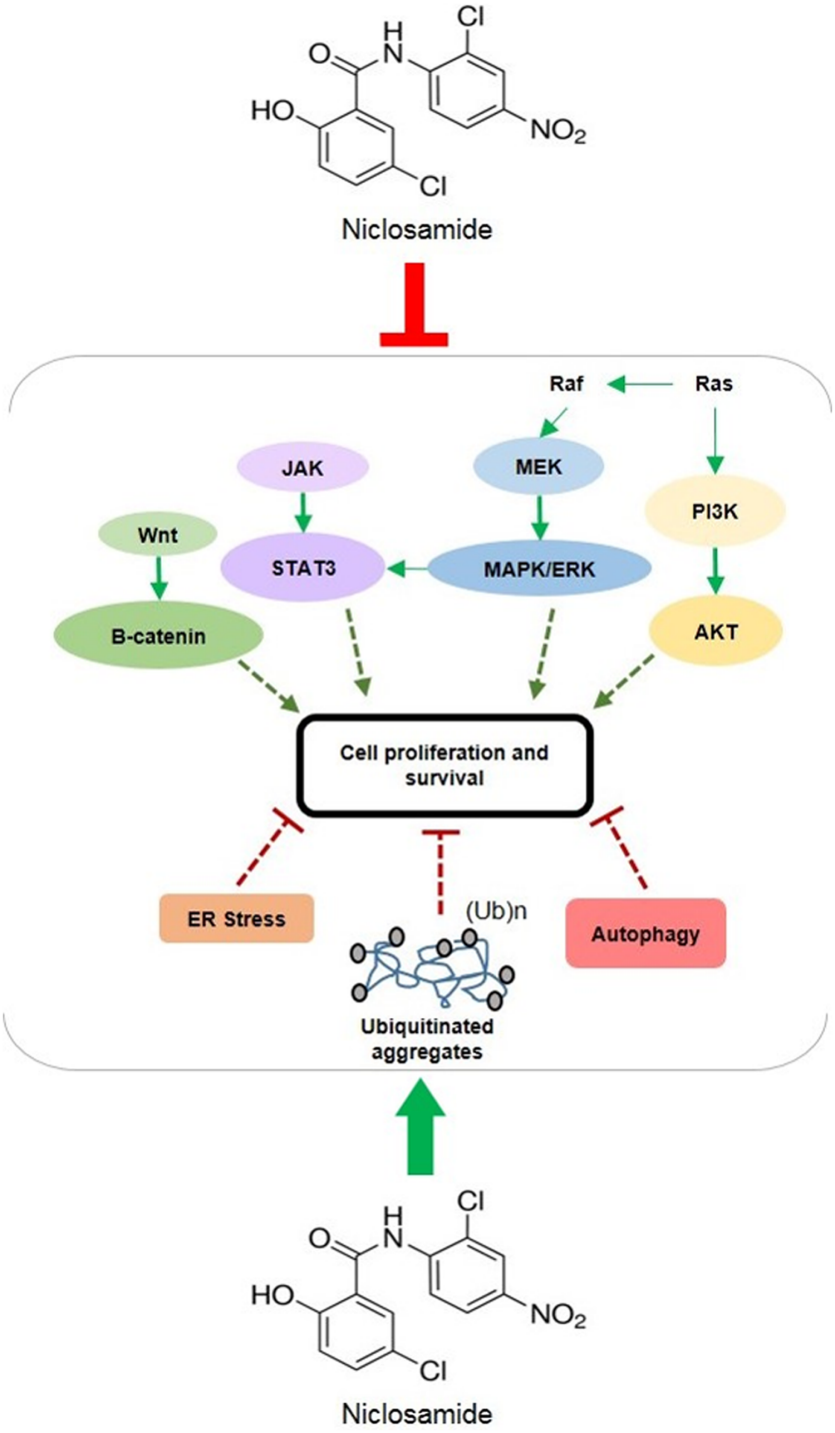
Schematic representation of niclosamide-mediated molecular mechanisms within human glioblastoma U-87 MG cell line. Niclosamide simultaneously inhibited multiple pro-survival signal transduction pathways and it activated other major cellular responses that inhibit cell proliferation and survival and facilitate cell death. Green lines represent activation/promotion of molecular pathways and red lines represent inhibition of cell signaling.

The present study demonstrated that niclosamide potently induced protein ubiquitination, endoplasmic reticulum stress and autophagy in human glioblastoma cells and considerably disrupted multiple signal transduction pathways that have been reported to be associated with glioblastoma development (Fig 7). Wnt/β-catenin, MAPK/ERK, PI3K/AKT and STAT3 signaling are highly activated in glioma, so it is conceivable that simultaneous pharmacological inhibition of all these signaling pathways might be the most effective therapeutic strategy for the prevention and treatment of glioblastoma. As our findings show, niclosamide appears to inhibit all these pathways within U-87 MG cells, resulting in decreased cell proliferation and increased cell death. Therefore, it would be of great interest to identify the mechanism(s)-of-action against these targets. Although its exact mode of action as an anthelmintic drug has remained unclear, niclosamide is believed to owe its anti-parasitic activity to its ability to uncouple oxidative phosphorylation (OP), block glucose uptake, and inhibit respiration in anaerobic ATP production [58–59]. Structurally, niclosamide is very similar to dinitrophenol (DNP), an antiseptic and a pesticide that also was used as a diet aide in the 1930s, enabling it to uncouple OP in the mitochondria during electron transport from NADH to flavoprotein [60]. In fact, niclosamide has been reported to target mitochondria in cancer cells to induce cell cycle arrest, growth inhibition and apoptosis [15]. It is possible that the mitochondria within glioblastoma cells are one of the targets of niclosamide, given that niclosamide has demonstrated a proton carrier mode-of-action independent of any protein target [61]. Niclosamide might cause an inward flux of protons through the mitochondrial membrane in glioblastoma cells and thereby dissipate proton gradients across the membrane to impair mitochondrial functions, which, in turn, would interfere with multiple pro-survival signaling pathways. A similar explanation might also apply to the cell membrane: niclosamide might target the cytoplasmic membrane of U-87 MG cells and induce intracellular acidification to disrupt pH homeostasis [41]. In fact, this hypothesis is consistent with some recent observations that intracellular acidification was able to induce protein ubiquitination in yeast [62]. Furthermore, it also is possible that niclosamide may target specific protein receptors on the surface of the cell, receptors that may be required for triggering the major signaling pathways.

## Conclusion

The present investigation demonstrates the tumor suppressive properties of niclosamide in human glioma U-87 MG cells. Niclosamide treatment simultaneously inhibited multiple key cell signaling pathways that are involved in cell proliferation and survival and it resulted in the initiation of ER stress, autophagy, and apoptosis. Even more interestingly, niclosamide acted like a proteasome inhibitor and markedly induced expression of abundant ubiquitinated proteins in U-87 MG cells, which mostly likely contributed to its effectiveness in suppressing cell survival and promoting cell death. It is rare to find a drug, especially one that has been established as safe for human use, which can target so many intracellular pathways involved in carcinogenesis. Overall, these results suggest that niclosamide may be a novel potential therapeutic candidate for the treatment of glioblastoma.

## Supporting information

S1 Fig Effect of niclosamide on protein ubiquitination in human U-118 malignant glioblastoma cell line. The human U-118 MG cell line was purchased from ATCC (Manassas, VA) and were cultured in DMEM medium containing 10% FBS at 37°C, 5% CO_2_. Cell lines were treated with the indicated concentrations of niclosamide for 24 h and then total cell lysates were collected. Protein was resolved by SDS-PAGE and immunoblotted with antibody specific to ubiquitin. β-Actin was utilized as a loading control.
(TIF)

S2 Fig Effect of niclosamide on protein ubiquitination in human cell lines. Cell lines were purchased from ATCC. BJ skin fibroblast cells (A) were cultured in EMEM containing 10% FBS; U-2 OS cells (B) were cultured in McCoy’s 5A medium containing 10% FBS; and MCF-7 (C) and MDA-MB-231 (D) cells were cultured in DMEM medium containing 10% FBS at 37°C, 5% CO_2_. Cell lines were treated with the indicated concentrations of niclosamide for 24 h and then total cell lysates were collected. Protein was resolved by SDS-PAGE and immunoblotted with antibody specific to ubiquitin. β-Actin was utilized as a loading control.
(TIF)

S3 Fig Effect of niclosamide on proteasome activity. U-87 MG cells were treated with the indicated concentrations of niclosamide for 24 h. 20S proteasome activity was quantified by a colorimetric assay (Cayman Chemical Company, Ann Arbor, MI) read at 480 nm and normalized by cell number. Data represent the mean ± S.E.M of at least three independent experiments.
(TIF)

S4 Fig Data set for statistical analysis of cell viability following niclosamide treatment (Fig 1). (XLSX)

S5 Fig Data set for statistical analysis of western blot densitometry (Fig 2). (XLSX)

S6 Fig Data set for statistical analysis of proteasome activity following niclosamide treatment (S3 Fig). (XLSX)
