# Supplementary figures and images for: Niclosamide induces protein ubiquitination and inhibits multiple pro-survival signaling pathways in the human glioblastoma U-87 MG cell line
Source: PLoS One. 2017 Sep 6;12(9):e0184324. doi: 10.1371/journal.pone.0184324 (PMC5587337; doi:10.1371/journal.pone.0184324)

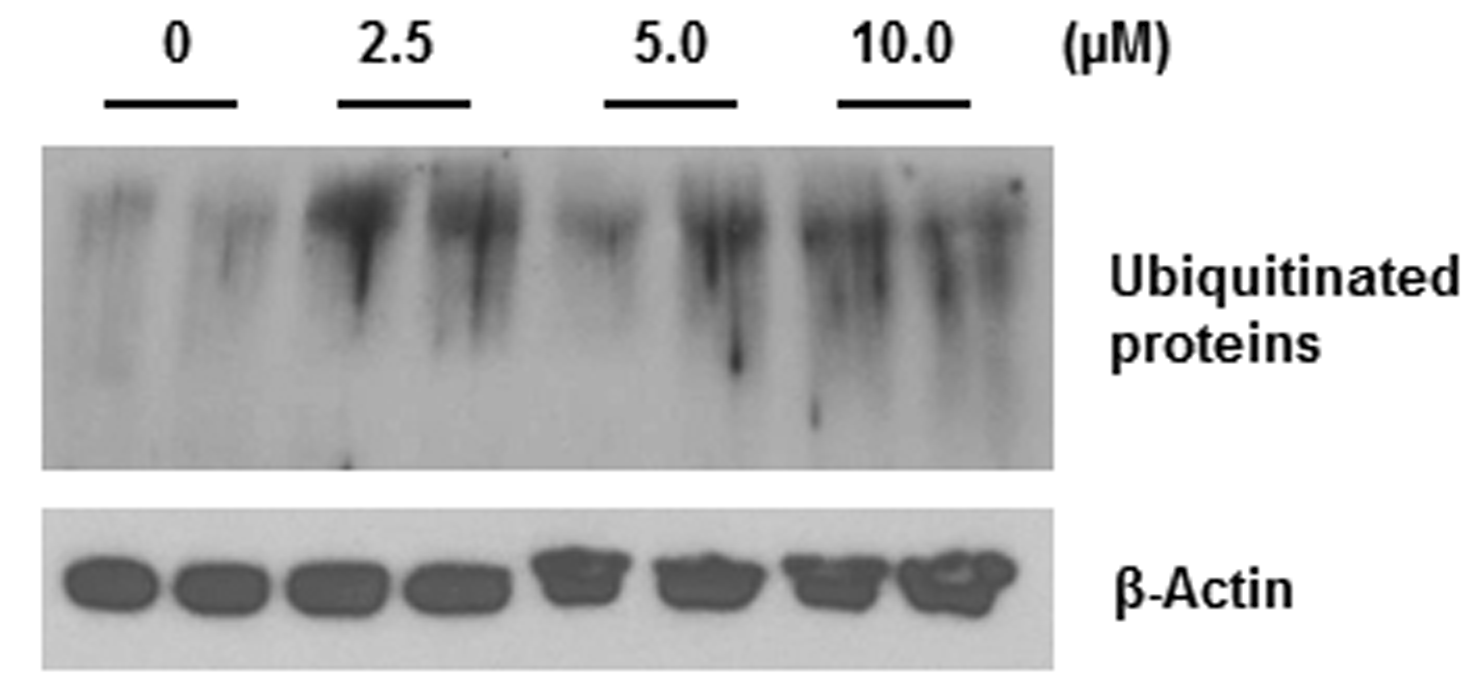

Supplement: S1 Fig — The human U-118 MG cell line was purchased from ATCC (Manassas, VA) and were cultured in DMEM medium containing 10% FBS at 37°C, 5% CO2. Cell lines were treated with the indicated concentrations of niclosamide for 24 h and then total cell lysates were collected. Protein was resolved by SDS-PAGE and immunoblotted with antibody specific to ubiquitin. β-Actin was utilized as a loading control. (TIF) [file pone.0184324.s001.tif]

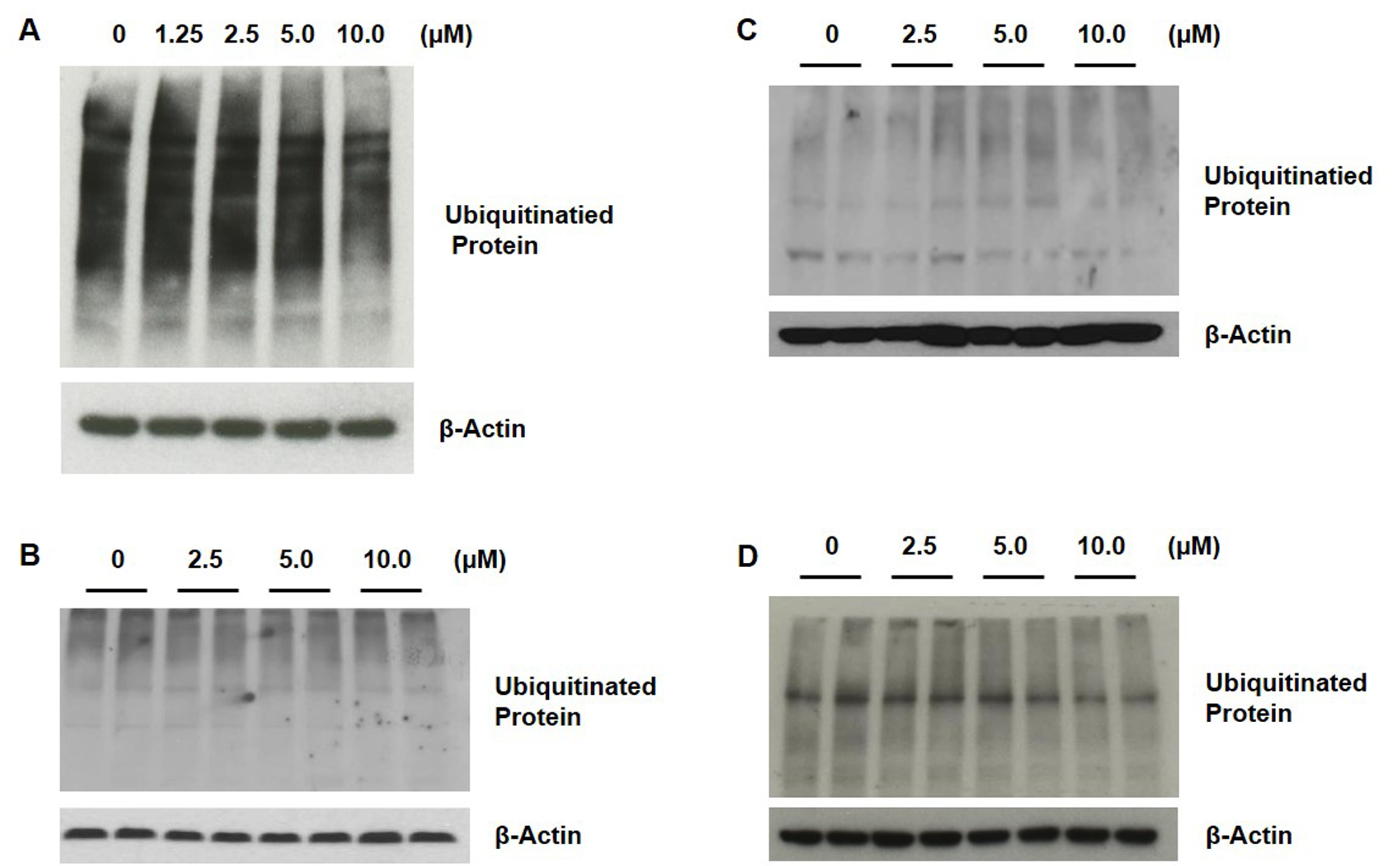

Supplement: S2 Fig — Cell lines were purchased from ATCC. BJ skin fibroblast cells (A) were cultured in EMEM containing 10% FBS; U-2 OS cells (B) were cultured in McCoy’s 5A medium containing 10% FBS; and MCF-7 (C) and MDA-MB-231 (D) cells were cultured in DMEM medium containing 10% FBS at 37°C, 5% CO2. Cell lines were treated with the indicated concentrations of niclosamide for 24 h and then total cell lysates were collected. Protein was resolved by SDS-PAGE and immunoblotted with antibody specific to ubiquitin. β-Actin was utilized as a loading control. (TIF) [file pone.0184324.s002.tif]

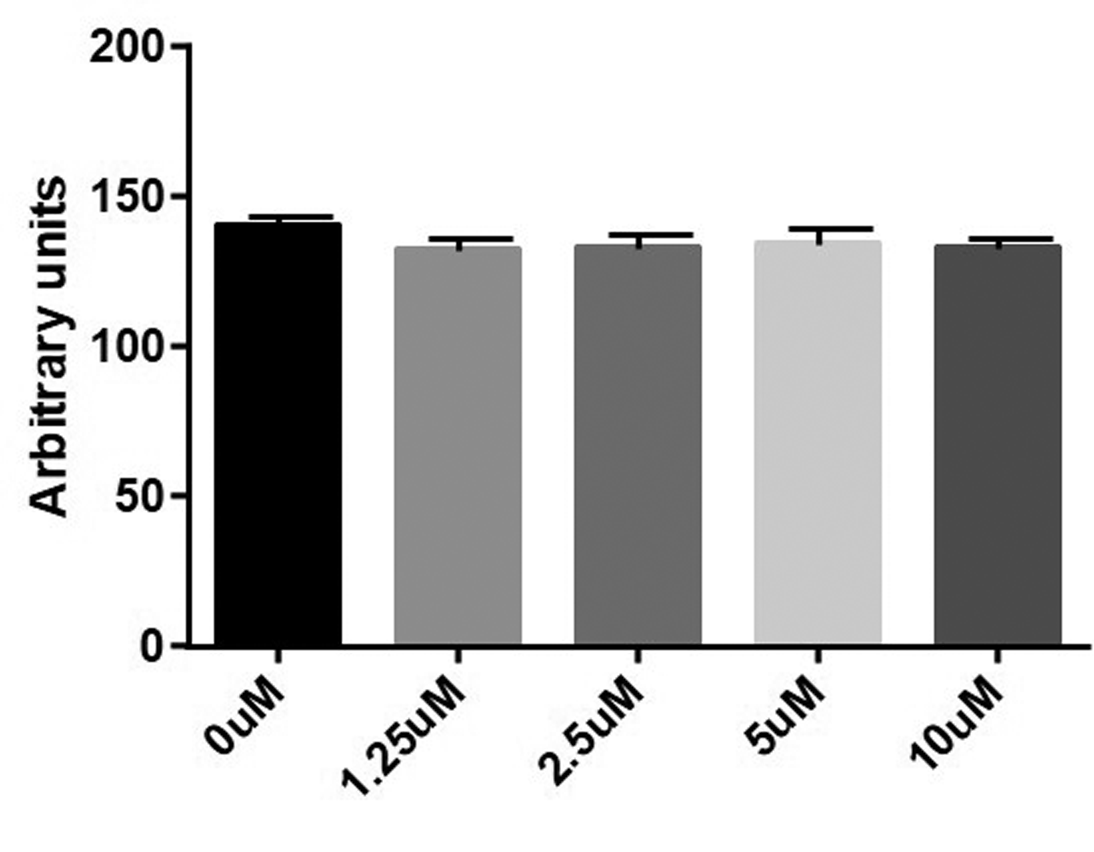

Supplement: S3 Fig — U-87 MG cells were treated with the indicated concentrations of niclosamide for 24 h. 20S proteasome activity was quantified by a colorimetric assay (Cayman Chemical Company, Ann Arbor, MI) read at 480 nm and normalized by cell number. Data represent the mean ± S.E.M of at least three independent experiments. (TIF) [file pone.0184324.s003.tif]
